# Acute-Phase Inflammatory Response to Single-Bout HIIT and Endurance Training: A Comparative Study

**DOI:** 10.1155/2016/5474837

**Published:** 2016-04-26

**Authors:** Felix Kaspar, Herbert F. Jelinek, Steven Perkins, Hayder A. Al-Aubaidy, Bev deJong, Eugene Butkowski

**Affiliations:** ^1^Department of Biotechnology, Institute for Biochemistry and Biotechnology, Technical University Braunschweig, 38126 Braunschweig, Germany; ^2^School of Community Health, Charles Sturt University, Albury, NSW 2640, Australia; ^3^Australian School of Advanced Medicine, Macquarie University, Sydney, NSW 2109, Australia; ^4^School of Medicine, University of Tasmania, Hobart, TAS 7001, Australia

## Abstract

*Objective*. This study compared acute and late effect of single-bout endurance training (ET) and high-intensity interval training (HIIT) on the plasma levels of four inflammatory cytokines and C-reactive protein and insulin-like growth factor 1.* Design*. Cohort study with repeated-measures design.* Methods*. Seven healthy untrained volunteers completed a single bout of ET and HIIT on a cycle ergometer. ET and HIIT sessions were held in random order and at least 7 days apart. Blood was drawn before the interventions and 30 min and 2 days after the training sessions. Plasma samples were analyzed with ELISA for the interleukins (IL), IL-1*β*, IL-6, and IL-10, monocyte chemoattractant protein-1 (MCP-1), insulin growth factor 1 (IGF-1), and C-reactive protein (CRP). Statistical analysis was with Wilcoxon signed-rank tests.* Results*. ET led to both a significant acute and long-term inflammatory response with a significant decrease at 30 minutes after exercise in the IL-6/IL-10 ratio (−20%; *p* = 0.047) and a decrease of MCP-1 (−17.9%; *p* = 0.03).* Conclusion*. This study demonstrates that ET affects the inflammatory response more adversely at 30 minutes after exercise compared to HIIT. However, this is compensated by a significant decrease in MCP-1 at two days associated with a reduced risk of atherosclerosis.

## 1. Introduction

Exercise and general physical activity are commonly associated with a healthy lifestyle and longevity as well as overall low levels of proinflammatory and high levels of anti-inflammatory cytokines [1].

Cytokines of the interleukin group, including interleukin 1*β* (IL-1*β*), interleukin 6 (IL-6), and interleukin 10 (IL-10), are key agents of the immune system and are involved in the systemic response to local inflammation [1]. Single exercise sessions of varying intensity and duration have been shown to induce systemic inflammatory responses similar to those associated with injury [2, 3].

The acute-phase response (APR) is the body's immediate response to inflammatory stimuli such as strenuous exercise and includes a complex mediator cascade aimed towards minimizing expansion of tissue damage and enabling recovery from proinflammatory processes. Exercise activates monocytes and macrophages with subsequent upregulation and expression of proinflammatory cytokines such as IL-1*β*, IL-6, and monocyte chemoattractant protein-1 (MCP-1) [4, 5]. MCP-1 levels, as well as those of the anti-inflammatory IL-10, have been shown to increase during and following exercise in order to contain inflammation and restore normal physiological function of the affected tissue [6–8]. IL-1*β* is also effective in the APR, proinflammatory cell activation, and stimulation of APR gene expression [4]. Despite its principal role in the APR, findings of exercise-induced IL-1*β* increases have been inconsistent [5]. Since IL-1*β* increases not only its own production but that of IL-6 and C-reactive protein (CRP) as well, elevated levels of this cytokine may have adverse effects in the context of inflammation containment [5, 9].

Physiological levels of C-reactive protein (CRP) are generally very low in healthy resting individuals but can increase up to 10,000 times during the APR [10]. Previous studies on the relationship between single bouts of exercise and the CRP response have revealed conflicting data as a number of studies demonstrated postexercise CRP level reductions, whereas others failed to do so [11, 12]. Further, the physiological levels of anabolic hormones such as insulin growth factor 1 (IGF-1), a pivotal mediator of muscle hypertrophy [13], appear not to change following a single bout of endurance or resistance exercise [2, 14].

High-intensity interval training (HIIT) is a type of workout regimen that has recently become popular due to its time effectiveness compared to traditional more time-consuming forms of endurance training (ET). HIIT yields similar training results such as an increase in mitochondrial enzyme activity, muscle oxidative capacity, and muscle glycogen content to conventional endurance training approaches by compensating the reduction in training volume with an increase in exercise intensity [8, 15–17].

Although the effects of single bout ET on inflammation have been extensively reported, studies on inflammatory responses to HIIT either investigated the systemic responses to repeated sessions [6] or examined HIIT in isolation [8] or in combination with other interfering stress factors including additional training volume and cumulative fatigue [18]. The overall consensus of these studies was that low-volume HIIT appears to lead to a similar inflammatory response to ET and supplies a sufficient training stimulus to generate significant physiological adaptations comparable to those achievable with endurance training in nonoverreaching individuals.

To gain a better understanding of the acute and midterm inflammatory responses to single exercise sessions and whether these may be related to the type of exercise, we investigated the effect of single-bout HIIT and ET on five inflammatory cytokines and IGF-1 in a group of young untrained adults. We hypothesize that HIIT may cause a different inflammatory response compared to ET due to its larger impact on heart rate and highly exhaustive character.

## 2. Methods

All participants were informed of the testing and training procedures and the potential risks involved. Participation was fully voluntary and participants could withdraw from the study at any time. Written informed consent was obtained prior to study commencement. All participants were instructed to abstain from exercise for 48 h preceding and following the exercise intervention. Consumption of alcohol and caffeine was prohibited for the duration of the study. This study was approved by the Human Research Ethics Committee (Charles Sturt University, Albury, Australia), protocol number 2014/161.

Nine healthy university students (Table 1) were recruited from student lectures two weeks prior to testing. Inclusion criteria were age 18–30 years, body mass index 18–30 kg·m^−2^, blood pressure 90/60–140/90 mmHg, not taking any medications or supplements including anti-inflammatory drugs, not currently diagnosed with a chronic health condition, nonsmoking, and being untrained (not training more than once per week at high or moderate intensity). Prior to the exercise bouts, participants were screened to ensure that they fit the inclusion criteria and that it was safe for them to undertake a high-intensity exercise program in accordance with the Exercise and Sports Science Australia Adult Pre-Exercise Screening Tool (2011) stages 1 and 2.

After completing the preliminary screening, participants were familiarized with the equipment used for the exercise protocols. Participants were randomly assigned to perform either the ET or the HIIT protocol first at least three days following the familiarization. The ET and HIIT sessions were performed at least seven days apart and in the morning (between 8 a.m. and 12 at noon). This 7-day washout period between the sessions was applied to ensure cytokine levels had returned to baseline before beginning the second intervention based on results by Ostrowski et al. [3], who described 5 days to be sufficient for cytokine levels to normalize.

The ET and HIIT sessions consisted of single supervised morning exercise sessions performed on an air-braked cycle ergometer (Wattbike Ltd., Nottingham, UK).

The HIIT session involved 2 min warm-up at <50 watts followed by 6 sets of 30 s of all-out supramaximal intensity cycling at the participant's respective self-selected gearing. Between sets participants completed a 4 min recovery period in which they either rested or cycled below 30 watts.

The ET session consisted of 45 min of ergometer cycling at a moderate intensity, which was calculated at 62.5% of maximal heart rate. Maximum heart rate was calculated according to the formula suggested by Tanaka et al. (208 − 0.7·age) [19]. Heart rate was measured by a heart rate monitor (RS800CX; Polar Electro Ltd.) with chest strap. Participants were able to monitor their current heart rate and were asked to maintain constant exercise intensity at their calculated heart rate.

Baseline blood samples were taken after 5 min of rest in a seated position on the day of the first intervention. Postexercise blood samples were taken 30 min and 2 days after completion of the ET and HIIT protocol. At each time point, 20 mL of blood was drawn from the antecubital vein in two 10 mL EDTA-tubes, centrifuged for 15 min at 800 g and 4°C. The plasma was transferred into 2 mL Eppendorf tubes (Eppendorf AG, Germany) and stored at −80°C until analysis (within 4 months).

Each sample was analyzed in duplicate using commercially available sandwich ELISA kits (http://www.elisakit.com/, Scoresby, VIC) in compliance with the supplier's instructions. According to the information provided by the manufacturer, the lower limit of quantification of the assays was less than 1 pg·mL^−1^ for IL-1*β*, less than 5 pg·mL^−1^ for IL-6, IL-10, and IGF-1, and less than 10 pg·mL^−1^ for MCP-1. The intra- and interassay coefficient of variance was <10% for all assays. The Lot numbers were IL-1*β*: #P-150702; IL-6: #L-150422; IL-10: #150409; IGF-1: #150612; MCP-1: #P-150417. The optical density at 450 nm was measured with a Multiskan FC Microplate Photometer (Thermo Fisher Scientific Inc., Waltham, MS).

Serum CRP levels and cholesterol profile were provided by Dorevitch Pathology Laboratory, Albury, NSW. Blood glucose levels were measured by BGL meters (Hoffmann-La Roche, Basel, Germany) from finger blood samples.

Data were analyzed with Microsoft Excel (Office 2010, Microsoft), SPSS (Version 22, IBM Inc.), and S-Plus 8 (TIBCO, Seattle, Washington). As participant numbers were below 10, the Wilcoxon signed-rank tests were applied to determine whether there were significant changes in the biomarker levels for post-ET and post-HIIT and between ET and HIIT. Correlation analyses were performed with Origin (OriginLab, Origin ProLab 9) and SPSS (SPSS v20, IBM). A *p* value < 0.05 was considered as significant. To determine the association between CRP, IL-6, and IL-1*β*, the synergistic model recommended by Ganter et al. was used [9].

Descriptive statistics for continuous variables were calculated in Excel and are expressed as mean ± SD. Immunoassay data are presented as mean ± standard error.

## 3. Results

Data from two of the initial nine participants were excluded from the analyses. One participant could not complete the HIIT protocol. Hemolysis impeded cytokine measurements of the second participant's samples.

There were no significant changes in the plasma levels of CRP, IL-1*β*, IL-6, IL-10, and IGF-1 from baseline to either 30 min or 2 days after the intervention (Table 2). However, there was a significant decrease in the IL-6/IL-10 ratio from baseline to thirty minutes after ET (−20%; *p* = 0.047). MCP-1 concentrations also decreased significantly from baseline to 2 days after ET (−17.9%; *p* = 0.03). These changes reflected at the 30 min mark where a trend towards a significant difference between the post-ET and post-HIIT change in CRP (ET 3.29 ± 1.01 > HIIT 2.07 ± 0.69; *p* = 0.1) and IL-6 (ET 27.25 ± 19.38 < HIIT 37.76 ± 19.49; *p* = 0.09) was observed.

A significant correlation between CRP and a calculated score based on IL-1*β* and IL-6 levels (Pearson *R* = 0.63; *p* = 0.01) was also observed. The response to the two cytokines is cooperative or synergistic and was calculated using normalized values of all patients from all points in time and an appropriate model for the synergistic action of IL-6 and IL-1*β* as suggested by Ganter et al. [9].

## 4. Discussion

This is the first study to directly compare the inflammatory response to single-bout ET and HIIT in the same cohort as well as investigate acute-phase response and medium-term response of cytokines to single-bout HIIT and ET. Our cohort was comparable in numbers to previous publications but used a repeated-measures design to obtain a better statistical power.

Endurance training reduces MCP-1 levels and has been associated with a reduction in the development of atherosclerosis, metabolic syndrome, and diabetes by acting on visceral fat reserves. MCP-1 has also been shown to be reduced following moderate levels of exercise in heart failure patients [20, 21]. Therefore, we sought to determine whether MCP-1 is reduced in young adults undertaking HIIT and whether this differs to results reported for ET. MCP-1 concentrations decreased after both training sessions and significantly decreased two days after ET. Zwetsloot et al. [8] showed that MCP-1 levels were significantly elevated immediately after exercise and Sugama et al. [22] demonstrated a high flush-out rate of this protein into urine. Therefore, our measured decreased levels may be lower following ET and HIIT due to the washout effect. However, the decreased MCP-1 levels two days after ET and after HIIT may also be due to a long-term decline in oxidative stress [23]. MCP-1 levels continued to decrease further following HIIT, which may be due to a more effective reduction in oxidative stress in addition to reported increased levels of lactic acid following HIIT [24, 25]. In the context of beneficial effects of single-bout ET and HIIT, one major health benefit of maintaining generally low levels of MCP-1 is the reduction of risk of cardiovascular disease and diabetes [26].

Our initial hypothesis that inflammatory responses would be different following single-bout ET versus single-bout HIIT was confirmed by a trend towards a greater decrease in the proinflammatory CRP and an increase in IL-6 levels following HIIT at thirty minutes following exercise. Neither training protocol appeared to cause significant acute changes of any single inflammatory cytokine possibly due to the relatively short training period (HIIT: 25 min, ET: 45 min). Previous investigations with shorter single-bout exercise duration (30 min) and lower intensity (50% maximal oxygen uptake) reported a comparable inflammatory response [12].

Previous work by Kasapis and Thompson [27] reported consistent increases in CRP levels after very strenuous exercise including marathons. The CRP decrease after HIIT in the present study is in agreement with the findings of Hovanloo et al. [6] who demonstrated a small CRP decrease 48 h after completing six HIIT sessions at a less strenuous level compared to Kasapis and Thompson.

A slight post-HIIT increase in IL-6 levels, which is in agreement with much of the literature, was observed [3, 8, 28]. These nonsignificant increases may be due to the short duration or lower exercise intensity, which did not affect muscle physiology enough to lead to a significant increase in IL-6 levels [29]. Single-bout ET and HIIT therefore have minor effects on muscle tissue integrity that nonetheless may lead to a greater reduction in CRP levels following HIIT by IL-6 acting as proinflammatory cytokine [1].

IL-6 is also the main inducer of CRP transcription but requires the presence of sufficient IL-1*β* in this regard. Although the presence of IL-6 by itself significantly raises CRP transcription, maximal expression only occurs when both cytokines are active [9]. Since IL-1*β* levels decreased after HIIT, which is in agreement with previous findings by Zwetsloot et al. [8], we employed a mathematical model to simulate a synergetic action of IL-6 and IL-1*β* on CRP expression as previously suggested by Ganter et al. [9]. The regulatory role of IL-1*β* in this context helps to explain why post-HIIT CRP levels decreased in spite of elevated IL-6 levels in our study. However, future studies will be required to validate this model.

No significant change in IL-10 levels was observed, although a slight decrease was noted, which is in agreement with the findings of Hovanloo et al. [6] and Zwetsloot et al. [8] regarding HIIT. Our findings for the ET intervention are inconsistent with the literature as most studies reported marked postexercise increases of IL-10 in contrast to our study showing no effect [22, 30]. However, these followed much more exhausting bouts of exercise. The recent study by Markovitch et al. [12], which resembles our ET protocol more closely, did not detect a significant IL-10 change. The observed decrease of plasma IL-10 levels could be attributed to a similar mechanism found for the MCP-1 decrease as Sugama et al. [22] also reported a rapid increase of IL-10 in the urine 1.5 h after exercise.

Although mean IGF-1 levels decreased after both ET and HIIT, there was no significant change to be noted. The findings are consistent with the results by Nindl et al. [14] who demonstrated that IGF-1 levels do not increase or decrease significantly after moderate or long duration aerobic or anaerobic exercise. The present study adds to this body of evidence and shows that circulating total IGF-1 levels do not change significantly after single bouts of ET or HIIT.

Regular aerobic exercise such as ET improves general health. HIIT, however, can produce similar results to ET including muscle oxidative capacity and mitochondrial enzyme activity, but in addition to providing various cardiometabolic benefits HIIT is also very time-efficient [17]. The lack of large-scale post-HIIT or post-ET inflammatory responses indicates that a regimen of two to three HIIT or ET sessions per week may noticeably aid in improving general health and fitness such as body fat percentage, maximal oxygen uptake, and cardiovascular endurance while exerting relatively little stress on the immune system in untrained and healthy young adults [16].

## 5. Conclusions

The novel findings of the present study are that (1) there was no significant difference between HIIT and ET in regard to individual cytokine responses and (2) IL-6 may be acting as an anti-inflammatory cytokine that leads to a greater decrease in CRP following HIIT and similar to ET a decreased MCP-1. These results support the hypothesis that HIIT may present a valid alternative to ET for individuals that are short on time or personally prefer a HIIT-type regimen to achieve equivalent overall health benefits to ET.

## Figures and Tables

**Table 1:** Subject characteristics.

Age (yr)	20.9 ± 0.9
Gender, M/F	1/6
Weight (kg)	69.5 ± 6.9
Height (cm)	169 ± 6.0
Body mass index (kg·m^−2^)	24.4 ± 2.3
Blood glucose level (mmol·L^−1^)	5.1 ± 0.8
SBP (mmHg)	116.7 ± 7.2
DBP (mmHg)	70.4 ± 4.7
HDL (mmol·L^−1^)	1.4 ± 0.2
LDL (mmol·L^−1^)	2.6 ± 0.8
Total cholesterol (mmol·L^−1^)	4.5 ± 1.0
Triglycerides (mmol·L^−1^)	1.1 ± 0.3

Data is presented as mean ± SD.

SBP: systolic blood pressure.

DBP: diastolic blood pressure.

HDL: high density lipoprotein.

LDL: low density lipoprotein.

**Table 2:** Blood marker data prior to and 30 min and 2 days after ET and HIIT.

	CRP^+^	IL-1*β*	IL-6	IL-10	MCP-1	IGF-1	IL-6/IL-10
Preexercise	3 ± 1	42.3 ± 5	30.4 ± 15	113.3 ± 24	224.8 ± 17	345.3 ± 70	0.3 ± 0.1
ET							
30 min	3.3 ± 1^#^	38.6 ± 5	27.3 ± 19^#^	109.2 ± 36	187.2 ± 28	302.4 ± 47	0.3 ± 0.1^#,*∗*^
2 days	2.92 ± 1.07	45.36 ± 5.10	33.94 ± 21.61	97.28 ± 28.80	184.68 ± 17.36^*∗*^	308.80 ± 58.24	0.50 ± 0.29
HIIT							
30 min	2.1 ± 0.7^#^	37.5 ± 4	37.8 ± 19^#^	97.5 ± 25	215.5 ± 25	299 ± 34	0.41 ± 0.16^#^
2 days	1.9 ± 2	39.5 ± 6	38.9 ± 23	82.9 ± 37	165.4 ± 22	256.2 ± 77	0.55 ± 0.2

Results are presented as mean ± SE.

^+^CRP concentrations are given in mg·L^−1^; all other concentrations are given in pg·mL^−1^.

^#^Trend towards significance at 30 min after exercise between ET and HIIT for CRP (*p* = 0.1), IL-6 (*p* = 0.09), and IL-6/IL-10 ratio (*p* = 0.08).

^*∗*^Significant changes for MCP-1 preexercise to 2 days after ET (*p* = 0.03) and for IL-6/IL-10 ratio for preexercise to 30 min after ET (*p* = 0.047).

## Additional Points

Practical implications are as follows:

- Lifestyle-induced sedentariness is a major contributor to obesity and diabetes in western societies and efforts to increase general physical activity are mostly unsuccessful. Therefore, various exercise protocols should be assessed.
- The present study shows that there is no significant difference between the inflammatory responses to conventional endurance training and high-intensity interval training and that neither protocol caused severe acute cytokine increases.
- Time-efficient high-intensity interval training, therefore, should be considered a valid alternative to conventional training approaches to improve general health and fitness.
